# *Implementation Science* six years on—our evolving scope and common reasons for rejection without review

**DOI:** 10.1186/1748-5908-7-71

**Published:** 2012-07-27

**Authors:** Martin P Eccles, Robbie Foy, Anne Sales, Michel Wensing, Brian Mittman

**Affiliations:** 1 Institute of Health and Society, Newcastle University, The Baddiley-Clark Building, Richardson Road, Newcastle upon Tyne, NE2 4AX, UK; 2 Leeds Institute of Health Sciences, Charles Thackrah Building, University of Leeds, 101 Clarendon Road, Leeds, LS2 9LJ, UK; 3 VA Inpatient Evaluation Center, Ann Arbor VA HSR&D Center of Excellence, Ann Arbor, MI, USA; 4 Scientific Institute for Quality of Healthcare, Radboud University Nijmegen Medical Centre, Nijmegen, Netherlands; 5 VA Center for Implementation Practice and Research Support, VA Greater Los Angeles Healthcare System, 16111 Plummer Street, Sepulveda, CA, 91343, USA

## Abstract

*Implementation Science* has been published for six years and over that time has gone from receiving 100 articles in 2006 to receiving 354 in 2011; our impact factor has risen from 2.49 in June 2010 to 3.10 in June 2012. Whilst our article publication rate has also risen, it has risen much less slowly than our submission rate—we published 29 papers in 2006 and 134 papers in 2011 and we now publish only around 40 % of submissions. About one-half of submitted manuscripts are rejected without being sent out for peer review; it has become clear that there are a number of common issues that result in manuscripts being rejected at this stage. We hope that by publishing this editorial on our common reasons for rejection without peer review we can help authors to better judge the relevance of their papers to *Implementation Science*.

## Background

*Implementation Science* has been published for six years and over that time has gone from receiving 100 articles in 2006 to receiving 354 in 2011; our impact factor has risen from 2.49 in June 2010 to 3.10 in June 2012. Whilst our article publication rate has also risen, it has risen much less slowly than our submission rate—we published 29 papers in 2006 and 134 papers in 2011 and we now publish fewer than 40 % of submissions. About one-half of submitted manuscripts are rejected without being sent out for peer review; it has become clear that there are a number of common issues that result in manuscripts being rejected at this stage. We set these out in order to help authors considering submitting to *Implementation Science*. They are summarized in Table 1 and addressed in the remainder of this article. In establishing and applying the criteria in Table 1, we endeavor to maintain consistency in our decisions irrespective of the clinical focus, approach to healthcare delivery, or setting involved in the manuscript. We are aware that we do not necessarily get it right all of the time; authors can help us make the right decisions on their manuscripts by making clear statements about, for example, the appropriateness of their study design and what the article adds to current knowledge or methodology.

**Table 1:** Summary of issues that influence the likelihood rejection without review of articles submitted to Implementation Science

**Issue**	**Likely to be accepted**	**Likely to be rejected**
Field of interest	Healthcare and public health	Anything else
Effectiveness studies	Evaluating the introduction of an intervention/evidence-based practice of known effectiveness	Evaluating the effectiveness of a clinical, organizational, public health, or policy intervention
Outcome	Health or health-related	Anything else
Implementation	Researching implementation	Doing implementation
Validity	Maximizes internal and external validity as appropriate in the chosen study designs	
Patient decision aids	Evaluations of the introduction of patient decision aids (of known effectiveness) into healthcare care settings; involvement of healthcare providers	Initial development or pilot testing of patient decision aids
Implementation (Knowledge Translation) direct to patients	Outcomes referring to evidence-based practice with some involvement of healthcare providers	Other types of outcomes
Intervention development reports	Prepared and submitted prior to the reporting of the effectiveness of the intervention	*Post hoc* submission
	Going to be, (robustly) evaluated	Not going to be (robustly) evaluated
Process evaluation	Submitted contemporaneously with or following report of intervention effectiveness	Process evaluations submitted in advance of the conduct of the main effectiveness analysis (it cannot be clear if they are explaining an effect or the absence of an effect)
	Process evaluations that take account of the main evaluation outcomes	Process evaluations that do not take account of the main evaluation outcomes
Pilot studies	If appropriate criteria for conduct	No justification for conduct
	If appropriate degree of inference	Overclaim on basis of results
	If there are plans for further evaluation	
Protocols	Been through (inter)national level peer review as part of their funding	Not been through national level peer review as part of their funding
	Received ethics review board approval	Not received ethics review board approval
	Submitted prior to data cleaning or analysis	Have begun data cleaning or analysis (may not apply to some qualitative studies)

### Journal scope

We receive a number of manuscripts that are not within our scope. Some are far outside our scope (*e.g.*, evaluations of novel clinical interventions), but others are more difficult to judge (*e.g.*, evaluations of novel service delivery methods). As one part of resolving a number of these more difficult or marginal issues we have, in discussion with our Senior Advisory Board and Editorial Board, refined our scope, particularly in relation to organizational, policy, and population-focused interventions.

This clarified a number of points:

· Our enduring field of interest is healthcare and evidence-based healthcare practice; we are interested in studies that report findings that would be of interest to a healthcare policy or decision maker, and in which there is a plausible connection, even distally, to health outcomes for patients;

· Our continuing principal interest in implementation strategies aimed at clinicians or clinical teams, and their organisations including strategies that imply a more active role for patients (see below);

· Clearer specification of our interest in the study of evidence-based organisational strategies as a method of introducing evidence-based healthcare;

· Clarification of the eligibility of public health interventions involving healthcare or healthcare professionals: public health papers that are evaluating the effectiveness of the introduction of health-related practices (of known effectiveness) are within our scope;

· Our interest in publishing generalizable findings (see below);

· Our exclusion of studies that are primarily concerned with establishing the effectiveness of a clinical or organisational intervention rather than the effectiveness of its implementation.

### Our revised scope is

‘*Implementation Science* is an open access, peer-reviewed online journal that aims to publish research relevant to the scientific study of methods to promote the uptake of research findings into routine healthcare in clinical, organisational, or policy contexts.

‘Biomedical, social science, organisational, and managerial research constantly produce new findings—but often these are not routinely translated into healthcare practice. Implementation research is the scientific study of methods to promote the systematic uptake of proven clinical treatments, practices, organisational, and management interventions into routine practice, and hence to improve health. In this context, it includes the study of influences on patient, healthcare professional, and organisational behaviour in either healthcare or population settings.

‘The lack of routine uptake of research findings is strategically important for the development of healthcare because it clearly places an invisible ceiling on the potential for research to enhance health. Further, it is scientifically important because it identifies the behaviour of healthcare professionals and healthcare organisations as key sources of variance requiring improved empirical and theoretical understanding before effective uptake can be reliably achieved.

‘Implementation science is an inherently interdisciplinary research area, and the journal is not constrained by any particular research method. *Implementation Science* wishes to publish articles of high scientific rigour using the most appropriate methods to produce valid, generalisable answers to research questions. As well as hosting papers describing the effectiveness of implementation interventions, *Implementation Science* provides a unique home for articles describing intervention development, evaluations of the process by which effects are achieved, and the role of theory relevant to implementation research. The journal is also interested in publishing articles that present novel methods (particularly those that have a theoretical basis) for studying implementation processes and interventions. We are also interested in receiving articles that address methodologically robust study of the de-implementation of ineffective clinical and organisational practices.

‘We welcome study protocols, but these will only be considered if the study has received ethics approval and been through external peer review via an established funding body. We do not consider protocols for systematic reviews or protocols for quantitative studies that have begun data cleaning or analysis.’

Alongside elaborating some of the issues coming out of this process, we also take this opportunity to address a number of common specific content and methodological issues that frequently lead us to reject a paper.

### Is it implementation research?

With our revised scope, our first major editorial question is ‘Is it implementation research?’ We expect a manuscript to deal with studying, as opposed to conducting, implementation—to be concerned with implementation science rather than implementation practice. Without a scientific question focused on implementation research, a manuscript will not be within our scope.

We accept six types of papers: Research, Systematic Review, Protocol, Methodology, Short Reports, and Debate. We no longer accept Meeting Reports. In general we expect Research, Systematic Review, Protocol, and Short Report manuscripts to focus on the formative (including research on implementation barriers, facilitators, and processes) or summative evaluation of the implementation of a defined, evidence-based, clinical practice, organizational, or policy intervention. Similarly, we would expect Methodology and Debate articles to focus on the methods for studying such implementation, or debate of key conceptual, theoretical, or methodological issues in the field. We understand that what is considered to be evidence-based and thus appropriate for implementation may vary, given the ongoing and complex debates about the nature of evidence, but authors need to make their arguments in relation to this very clear.

Our judgment is informed by how authors contextualize and situate their manuscript within the existing literature—whether or not the manuscript’s background section identifies and discusses the relevant implementation science literature (including systematic reviews) as appropriate, and whether or not the aims (and discussion) include an explicit statement about what the paper adds to existing knowledge, research, or research methods. We recommend that authors consider drafting a short paragraph that begins ‘Our study adds the following new information or knowledge to the existing literature…’ as a method of ensuring that this is clearly communicated.

There are two other scope issues that are linked—implementation strategies directed to patients without involvement of healthcare providers or the healthcare system, and decision aids.

### Implementation directed to patients

The Cochrane Effective Practice and Organisation of Care (EPOC) Review Group criteria offer a useful basis for our policy regarding patient-directed implementation; they allow patient mediated interventions defined as follows:

‘Interventions that aim to influence professional practice through patients are within the scope of EPOC. However, interventions that are solely aimed at changing the behaviour of consumers, such as lifestyle counseling, are not within EPOC's scope, unless both professional and patient behaviour is affected, for example interventions that aim to improve smoking cessation counseling by professionals where smoking cessation is the primary outcome measure, or interventions aimed at improving professionals' management of hypertension or diabetes where blood pressure or blood sugar control, respectively, is the primary outcome measure.’ (EPOC Taxonomy http://epoc.cochrane.org/information-specific-epoc-reviews accessed July 12 2012)

In general we adopt an EPOC-like position. However, we need some degree of flexibility. Consider a situation where a secondary care clinician and a policy maker say ‘we have a large problem with identification, diagnosis, and management of problems with continence in older women. We know they don’t present to primary care, and when they do primary care doctors don’t manage it.’ They mount a trial where they mail (evidence-based) guidelines on pelvic floor exercises directly to women over the age of 55 years. The trial shows an effect on care processes, including an increase in appropriate identification, diagnosis, and/or management by primary care clinicians. The policy maker then has an evidence-based guideline and an evidence-based implementation strategy that is policy relevant and generalizable to any area that has poor primary healthcare provision and a postal service.

The issue of women not presenting for care is a health promotion issue or a clinical issue depending on perspective. The issue of primary care doctors not dealing with it is an implementation issue. If the healthcare professional (primary care doctor in this case) is a barrier, then we can see a policy maker expressing interest in an effective patient-mediated implementation strategy. This does not mean that in another setting a policy maker would not want to address the issue by intervening with primary care doctors directly—but the result is still the implementation of a guideline with benefits in improved clinical processes and outcomes. If an author articulates *all* of these points, then we are more likely to consider such a study within scope.

We are interested in evaluations of the introduction of patient decision aids (of known effectiveness) into routine care settings. These studies will usually involve multiple routine care settings, usually with multiple healthcare professionals and unselected patient populations. We regard the initial development or pilot testing of patient decision aids as out of scope. Some emerging trends in shared decision-making research that go well beyond the application of decision aids in practice, such as implementation of inter-professional shared decision-making practice involving substantial change in health professional behavior and/or system of care, would likely be within scope.

### Generalisability

As well as being interested in internal validity, we are very interested in studies that produce clearly generalizable results (have high external validity), or that provide evidence of transferability across settings. We would like the readers of *Implementation Science* to be able to read a description of a study and, when they try and replicate it, have some *a priori* idea of what will happen as a consequence.

We do not impose methodological criteria for how authors might achieve this, but a variety of designs are available: pragmatic (cluster) randomized controlled trials (RCTs), interrupted time series studies, multiple case studies, case studies and qualitative research with a strong (explicit and *a priori*) theoretical underpinning.

### Intervention development reports

We welcome articles describing implementation intervention development in the context of an ongoing or subsequent evaluation of the effectiveness of the intervention. In general, implementation intervention development reports, like protocols, should be prepared and submitted prior to reporting the effectiveness of the intervention. If authors wished to publish an intervention development report after the publication of its effects are known, they would have to make a clear and strong argument for why such an intervention description merited publication and address why it had not been described *a priori*. To do otherwise risks authors modifying (consciously or not) their description of an intervention in the light of their knowledge of its effectiveness.

We receive a number of papers describing intervention development where the plan is to subsequently introduce the intervention without evaluation (*i.e.*, a case study) or a weak evaluation design that cannot rule out a multitude of alternative explanations for observed effects (*e.g.*, a single site after or before/after design). We do not accept these.

### Methodological and conceptual studies

We often receive manuscripts aiming to report novel methodological approaches or new conceptual frameworks relevant to implementation research. We welcome any such work that aims to advance our science. However, we often end up rejecting such submissions where it is unclear what the methodological development or (yet) another conceptual framework adds to existing literature. It is important that authors considering submitting such articles ensure that they have argued the reasons for choosing their method/theory/framework instead of the plausible alternatives.

### Process evaluations of interventions

In general, we encourage the submission of process evaluations—studies of what mediates or moderates the effects of an intervention. However, a process evaluation is useful only when reported alongside the implementation intervention it aims to explain. Whilst this can happen either in a main report paper or in a simultaneous paper, if they are in separate papers then ideally any journal should be considering both manuscripts. Most importantly, the editor needs to know what the main outcomes in the trial were in order to understand the measures used in the process evaluation.

We do not accept:

· Process evaluations submitted in advance of the conduct of the main effectiveness analysis, because it cannot be clear if they are explaining an effect or the absence of an effect;

· Process evaluations that do not take account of the main evaluation outcomes.

### Pilot studies

*Implementation Science* will consider appropriately designed and configured pilot studies. Our general principle is to use the same criteria that would be applied to a funding decision. For example, a small study would be useful if little or no evidence exists in a given area and the risk involved in a larger study is too great, and if the small study minimizes bias and assures basic levels of validity, even if the confidence interval around the results turns out to be large. If better evidence exists, then a small study will generally be of little value and will be difficult to justify as a contribution to existing knowledge.

An exception to this rule may be when a pilot is conducted with the explicit purpose of assessing feasibility and planning for a complex intervention that is expected to contribute to existing knowledge, as described in the UK Medical Research Council updated guidance on evaluating complex interventions [1], or in the Veterans Affairs Quality Enhancement Research Initiative (QUERI) framework for planning a national level implementation project [2]. In this case, if the goals of the pilot include the following, and provide potentially generalizable knowledge, we may be interested in publishing a report: development of a protocol for a complex intervention with description of acceptability, new techniques, specific issues of recruitment and consent; development of new measures; interesting methods or issues related to randomization; and sample size calculation, including estimation of intraclass correlation in the case of clustered data [3]. In most cases, we would expect reports of pilot studies to be relatively brief, and typically appropriate for the Short Report format.

If a manuscript describing a pilot study is being considered, then it is very important that the authors report it as such with due caution in relation to the provisional nature of the results and, in the context of a ‘successful’ pilot, preferably indicating how a subsequent study will draw from the pilot study.

### Qualitative studies

We frequently receive papers using qualitative methods for data collection and analysis. Many of these are of high quality, and we publish those that fit our scope and meet applicable criteria for quality and validity. However, we often find problems in the reporting or design quality of papers using qualitative methods, or in the clarity of their contribution to existing knowledge. Not infrequently, we receive papers that report on essentially content analysis of interviews, often without appropriate links to relevant theory or without contextualization. We routinely reject these.

We currently recommend use of the RATS guidelines for assessing minimal quality of reporting of qualitative research (http://www.biomedcentral.com/ifora/rats accessed 6 July 2012), which we also advise our reviewers to consider as they review qualitative papers. In addition to using RATS as a check prior to submission, however, we also encourage authors to read some of the literature that forms a backdrop to what is a very lively ongoing debate and discussion about what constitutes high-quality reporting of studies using qualitative methods [4,5]. We note in particular that high-quality reporting of research using qualitative methods requires some amount of reflection about the epistemology underlying the choice of methods, as well as clear and comprehensive description of the context or setting in which the study was conducted and in which the participants exist.

### Reporting guidelines

Our instructions to authors already state our support for reporting guidelines, but it is worth re-stating here because many authors seem not to use them. Where available, methodological assessment and adequacy of reporting should be judged against relevant reporting guidelines. Whilst there are many reporting guidelines (see http://www.equator-network.org/home/ accessed 6 July 2012), those of particular relevance to *Implementation Science* are (http://www.implementationscience.com/about):

· Trials—CONSORT, particularly the extension for cluster trials;

· Systematic review—PRISMA;

· Qualitative studies—RATS;

· Surveys—Reporting guidelines for survey research [6];

· Intervention reporting—http://www.implementationscience.com/content/4/1/40.

For RCTs and systematic reviews, respectively, CONSORT (with the extension for cluster trials) and PRISMA checklists should be completed and uploaded as an additional file for the editor to check, and authors should also include a flowchart as part of the manuscript. In addition, authors should make any amendments identified on the basis of completing the checklist.

Whilst CONSORT applies specifically to RCTs, authors of studies using other evaluative designs would be well advised to consult the reporting guidelines because many of the issues can, with benefit, be applied to non-randomised studies.

### Protocols

Our policy regarding publication of protocols has recently changed and, so again, whilst this is reflected in our journal web pages, it is worth re-stating here. The case for publishing protocols is most clearly made for trials. Although some of the ‘advertising’ arguments can apply to studies using other designs, the *a priori* statement of study outcome measures and analyses is important for trials. Many of the protocols we currently publish are for trials, and though we do publish protocols for study designs other than trials, this is currently under review. We would be interested in readers’ views on the publication of protocols for studies other than trials.

We accept (without further peer review) protocols that have been through (at least) national level, competitive peer review as part of their funding and that have received ethics review board approval or exemption. Protocols for programs of research may be an exception to this requirement and are considered on a case-by-case basis.

We do not accept:

· Protocols that have not been the subject of national or international level peer review as part of funding;

· Protocols for pilot studies—Because pilot studies are intended to lead on to subsequent, larger studies then there will be considerable overlap between the content of protocols for the two, and concerns about duplicate publication then arise. Authors should concentrate on publishing protocols for their subsequent, larger studies;

· Protocols for systematic reviews—We refer systematic review protocols to the new BMC journal, *Systematic Reviews*;

· Protocols that are submitted for studies (particularly cluster RCTs) where data cleaning and analysis have begun. Having a cut point like this is a common requirement of journals that publish trial protocols (in clinical trials, it is usually the end of patient recruitment) so that publication is a truly prospective event and the content of a protocol cannot be influenced (however unlikely this might be) by knowledge of the data. This may not apply to some qualitative studies but, in general, the intention is for a protocol to be published prior to any analysis in order to prevent bias.

Authors of trial protocols also need to have registered the study with an appropriate trial database and to complete an appropriate reporting guideline checklist (see section above on reporting guidelines).

## Conclusions

We hope that by publishing this editorial on our common reasons for rejection without peer review we can help authors to better judge the appropriateness of their papers to *Implementation Science*. We will continue to review and refine our policies as the journal and the research field continue to evolve, and welcome author and reader comments and debate to guide this review.

## Competing interests

The authors all hold editorial positions with Implementation Science.

## Authors’ contributions

MPE drafted the manuscript and revised it in the light of comments from the other authors. All authors read and approved the final manuscript.
